# Children's self‐reports of fear and pain levels during needle procedures

**DOI:** 10.1002/nop2.399

**Published:** 2019-10-15

**Authors:** Lena Hedén, Louise von Essen, Gustaf Ljungman

**Affiliations:** ^1^ Faculty of Caring Sciences, Work Life and Social Welfare University of Borås Borås Sweden; ^2^ Department of Women's and Children's Health Clinical Psychology in Healthcare Uppsala University Uppsala Sweden; ^3^ Department of Women's and Children's Health Pediatric Oncology Uppsala University Uppsala Sweden

**Keywords:** cancer, children, fear, needle, nurses, nursing, pain, self‐report

## Abstract

**Aim:**

The objective was to determine the levels of and potential relationships between, procedure‐related fear and pain in children.

**Design:**

Clinical based cross‐sectional.

**Methods:**

Ninety children aged between 7–18 years were included consecutively and self‐reported levels of pain and fear on a 0–100 mm visual analogue scales (VAS) when undergoing routine needle insertion into a subcutaneously implanted intravenous port following topical anaesthesia.

**Results:**

The needle‐related fear level was reported to be as high as the needle‐related pain level (mean VAS: 14 mm and 12 mm, respectively, *N* = 90). With fear as the dependent variable, age and pain were significantly associated and explained 16% of the variance. With pain as the dependent variable, fear was significantly associated and explained 11% of the variance. A post hoc analysis indicated that younger children reported their fear levels to be higher than their pain levels.

## 1 INTRODUCTION

Needle procedures in relation to disease and treatment are often experienced as frightening and painful for children. Painful procedures are a common problem for children during the cancer disease trajectory and are reported to be worse than both cancer pain and pain related to treatment side effects (Hedström, Haglund, Skolin, & von Essen, 2003).

### 1.1 Background

A relationship between fear and pain has been established, and it is known that fear can increase the experience of pain through psychological and physiological mechanisms (Huguet, McGrath, & Pardos, 2011; Rhudy & Meagher, 2003). Evidence suggests that fear plays an important role in the experience of pain during needle procedures (Uman et al., 2013). The experience of pain is modified by several factors such as earlier painful experiences, fear, child age and gender (Goodenough et al., 1999). A previous study from the parental perspective found that fear levels were higher than pain levels when topical anaesthesia was used during needle procedures in children (Hedén, von Essen, & Ljungman, 2016). This study adds the children's voice through self‐report of fear and pain during a needle procedure.

The most commonly reported fears in healthy children aged 5–16 years are animals, blood/injections and darkness (Meltzer et al., 2009). Fear is a normal reaction to threat or danger (e.g. separation from parents or darkness) which usually decreases with age (Ollendick, 1983). However, this does not always apply to fear in the medical context (Gullone, 2000). Both fear and pain can be learned and remembered from earlier experiences, (Anderzen Carlsson, Sörlie, Gustafsson, Olsson, & Kihlgren, 2008) and negative experiences during needle procedures such as injections may lead to needle fear in some children. This can result in avoidance of medical contexts and immunization non‐compliance (Taddio & McMurtry, 2015). Consequently, it is essential to consider fear when determining procedure‐related pain. Several pain‐reducing interventions exist, including topical anaesthesia before needle insertion and distraction. However, the needle procedure entails a short sharp pain and even when the skin is numbed by topical anaesthesia, many children experience needle procedures such as insertion of a needle in a subcutaneously implanted intravenous port as frightening, painful and/or distressing (Ljungman, Gordh, Sörensen, & Kreuger, 2000; Ljungman et al., 1996).

Our hypothesis is that fear may be as big problem as pain from a child's perspective during needle procedures involving topical anaesthesia. To the best of our knowledge, no previous study has explored the child's experience and the potential relationship between fear and pain during needle insertion in a subcutaneously implanted port in children with cancer. Our research group previously reported fear and pain levels in this situation from the parental perspective, concluding that parents report the level of fear to be higher than the level of pain in their children (Hedén et al., 2016). Therefore, it can be relevant to target the most suitable interventions during needle procedures to alleviate fear, pain and other important variables.

According to the hypothesis, the primary research question was “How high are the children's levels of fear and pain in needle procedures and how are they related?” The primary objective was to determine children's experience of the levels of and potential relationships between, procedure‐related fear and pain in a model using needle insertion in a subcutaneously implanted port under topical anaesthesia as the painful procedure. Secondary objectives were to determine any associations between fear and pain and the child's age, sex, time since last needle insertion and as a physiological stress response; cortisol changes levels in relation to fear or pain.

## 2 METHOD

A clinical based cross‐sectional design with child self‐report was used. The data reported in this paper were collected from 2006–2010 in a project investigating the effect of pharmacological interventions on fear and pain using randomized controlled trials in a paediatric oncology and haematology setting (Hedén, von Essen, Frykholm, & Ljungman, 2009; Hedén, von Essen, & Ljungman, 2011; Hedén, Essen, & Ljungman, 2014). In the present study, we explored the relationship between levels of fear and pain reported by children treated with placebo or study medication (midazolam/morphine/paracetamol/ibuprofen) in addition to standard care with topical anaesthesia.

### 2.1 Patients

**Figure 1:**
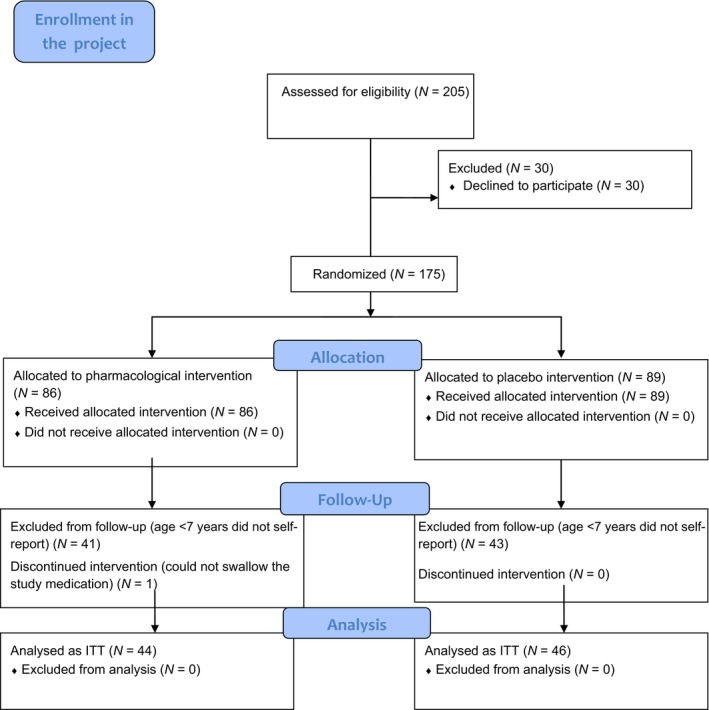
Flow diagram for study inclusion showing the 90 children analyzed on an intention to treat (ITT) basis

Figure 1 illustrates the flow of participants throughout the project, (Hedén, Essen, Frykholm, et al., 2009; Hedén et al., 2011, 2014) as recommended by the CONSORT statement (Schulz, Altman, & Moher, 2010). All children who did not self‐report were excluded from the initial 175 children, leaving reports from 90 children (35% girls) aged 7–18 years in the analysis. Patient characteristics are presented in Table 1.

**Table 1:** Patient background characteristics (*N* = 90)

Age in years, mean (*SD*)	13 (3.6)
Over/under 12 years	56/34
Girl/boy (%)	35/55 (39/61)
Diagnosis group	
Leukaemia/lymphoma	47
CNS	15
Other	28
Physical status, ASA^a^ I/II/III	7/75/7^b^
Weeks since diagnosis, mean (*SD*) median	45 (63) 20
Weeks since latest needle insertion, mean (*SD*) median	7 (18) 2

a ASA = American Society of Anesthesiologists Physical Status Classification System.

b 1 missing value.

Exclusion criteria in the whole project were (1) age <1 or ≥20 years, (2) moderate to severe pain assessed by a visual analogue scale (VAS) (>50 on a 0–100 mm scale) from causes other than the needle insertion (e.g. ongoing cancer pain), (3) fever >39°, (4) nausea, (5) a documented need for pharmacological sedation during needle insertion, (6) inability to understand Swedish and (7) concurrent treatment with, or hypersensitivity to, any of the pharmacological interventions (midazolam/morphine/paracetamol/ibuprofen).

Assessment of disease‐related pain or other type of pain not related to the procedure was made before inclusion, usually as part of daily care and documented in the patient chart. The patients in this study included both children and adolescents, but for simplicity they are all referred to in this article as “children.” The children all had multiple previous experiences of needle insertions.

### 2.2 Procedure

Written informed consent was obtained from parents/guardians, and oral informed assent was obtained from children. All participating children received standard care including Eutectic Mixture of Local Anesthetics (EMLA^®^, AstraZeneca) patch/cream for ≥60 min at the site of the needle insertion and were provided with information according to the usual routines. The same routine and material for needle insertion into the port were used during the entire study period. The children were allowed to lie down in their preferred positions during the needle insertion and were supported by their parents.

### 2.3 Evaluation

The children reported their fear and pain during an intravenous port needle insertion. These reports were made directly after the needle insertion. The children were asked: “How much pain/fear did you experience when the needle was inserted?” and responded on a 100 mm VAS with anchors at the extreme ends (no pain/fear to worst possible pain/fear). Higher scores represented more pain/fear. The VAS has previously been used both by our research group and by Pagé et al to assess children's pain and fear (Hedén, Essen, Frykholm, et al., 2009; Hedén, von Essen, & Ljungman, 2009; Hedén et al., 2011; Pagé et al., 2012). The stress response connected to children's procedure‐related pain has been assessed by others using cortisol levels in serum as a biochemical indicator of distress (Felt et al., 2000). Cortisol levels increase approximately 15–30 min after a stressful situation. The level normally varies over day and night and so the increase or decrease compared with baseline should be analysed, rather than the absolute level (Hanrahan, McCarthy, Kleiber, Lutgendorf, & Tsalikian, 2006). Thus, cortisol in serum was sampled at the time of the needle insertion and after 30 min, through the existing needle in the port in both instances. The difference between these two samples represented the change in cortisol level in the present study.

### 2.4 Statistical analysis

Descriptive statistics were used for background characteristics. A dependent *t* test was used to investigate potential differences between fear and pain levels, and Pearson correlation coefficients were used to describe bivariate associations between study variables. Multiple regression analyses with backward selection to minimize suppressor effects were used to investigate the effects on fear and pain of the child's age, sex, time since last needle insertion and change in cortisol level. For the purpose of analysis, change in cortisol level from needle insertion to 30 min after insertion was analysed (Table 2).

**Table 2:** Descriptive statistics of VAS (visual analogue scale 0–100 mm) fear levels, VAS pain levels and cortisol levels (mmol/l)

	*N*	Mean	Median	*SD*
Fear	90	14	5	22
Pain	90	12	4	15
Cortisol sample 1	83	183	159	137
Cortisol sample 2	83	159	147	122
Cortisol change^a^	83	−24	−12	54

a Sample 1: directly after needle insertion. Sample 2: 30 min later.

A medium‐sized relationship between the independent variables and the dependent variable (fear and pain respectively) was assumed with an alpha at 0.05 and a positive or negative beta at 0.20. According to the rule of thumb expressed by Tabachnick and Fidell (2007), it was calculated that 90 cases would be sufficient for five independent variables per dependent variable/regression (Tabachnick & Fidell, 2007). The calculated statistical power level was 0.80 for the multiple regression analysis. The residuals were normally distributed in the regression analyses for fear and pain, with Durbin–Watson values of 1.7 and 2.3, respectively. The regression models were used only to explore relationships between the different variables. Statistical analyses were performed using version 23.0 of the Statistical Package for the Social Sciences (SPSS; SPSS Inc.).

### 2.5 Ethical considerations

The study project was approved by the Regional Ethics Committee (2004:M‐362) and conducted according to the Declaration of Helsinki. The trials in the project are registered in the European Clinical Trials Database EudraCT (2004‐002378‐42; 2005‐002112‐17; 2005‐002110; 2005‐005645‐19).

Written informed consent was obtained from parents/guardians, and oral informed assent was obtained from children. The study information was adapted to child age level, both orally and in writing. The participants and guardians were also informed that they could interrupt their participation in the study at any time without explanation or consequences.

## 3 RESULTS

Data from 90 children aged 7–18 years were analysed (Table 1). The reported mean VAS fear level was 14 mm (*SD* 22), and the mean VAS pain level was 12 mm (*SD* 15) (*N* = 90). Mean cortisol values in serum were 183 mmol/l (*SD* 137) directly after needle insertion and 159 mmol/l (*SD* 122) 30 min later, giving a cortisol change level of −24 (see Table 2).

Bivariate correlations between fear and pain and background variables are presented in Tables 3 and 4. The fear level during needle insertion was associated with the pain level (*r* = .33, *p* < .01) and with child age (*r* = −.23, *p* < .05).

**Table 3:** Correlations between background variables and level of fear (*N* = 90)

Fear	Pearson correlation^a^
Sex, girl/boy	0.110
Age^b^	−0.233
Time since last needle insertion	−0.040
Cortisol change^c^	−0.015
Pain^d^	0.327

a Point‐biserial correction applied (SPSS 23).

b Correlation is significant at the <0.05 level (1‐tailed).

c Sample 1: directly after needle insertion. Sample 2:30 min later.

d Correlation is significant at the <0.01 level (1‐tailed).

**Table 4:** Correlations between background variables and level of pain (*N* = 90)

Pain	Pearson correlation^a^
Sex, girl/boy	0.046
Age	0.084
Time since last needle insertion	−0.134
Cortisol change^b^	−0.038
Fear^c^	0.327

a Point‐biserial correction applied (SPSS 23).

b Sample 1: directly after needle insertion. Sample 2:30 min later.

c Correlation is significant at the <0.01 level (1‐tailed).

There were no differences in age, sex, diagnostic group, American Society of Anesthesiologists' Physical Status Classification System (ASA) level, or time since last needle insertion between children who received pharmacological intervention and those who received placebo, or between children who participated and those who declined participation.

### 3.1 Fear as dependent variable

The child's age and pain explained 16% of the variance in fear (Table 5). There were no associations between fear and the child's sex, time since last needle insertion, or cortisol change level and fear in the multiple regression analysis.

**Table 5:** Multiple regression with fear as dependent variable (*N* = 90)

Model	Variables	Unstandardized coefficients		Standardized coefficients	*t*	*p*	adj *R* ^2^
		*B*	*SE*	Beta			
1	(Constant)	28.6	8.5		3.4	.001	.14
	Sex, girl/boy	5.0	4.6	0.1	1.1	.277	
	Age	−1.6	0.6	−0.3	−2.7	.008	
	Time since last needle insertion	−0.04	0.1	0.0	−0.4	.726	
	Cortisol change^a^	0.0	0.0	0.0	−0.3	.754	
	Pain	0.5	0.1	0.4	3.4	.001	
4	(Constant)	29.5	8.1		3.6	.000	.16
	Age	−1.6	0.6	−0.3	−2.7	.009	
	Pain	0.5	0.5	0.3	3.6	.001	

a Sample 1: directly after needle insertion. Sample 2: 30 min later.

### 3.2 Pain as dependent variable

Fear explained 11% of the variance in pain (Table 6). There were no associations between pain and the child's sex, age or time since last needle insertion during needle insertion and pain in the multiple regression analysis.

**Table 6:** Multiple regression with pain as dependent variable (*N* = 90)

Model	Variables	Unstandardized coefficients		Standardized coefficients	*t*	*p*	adj *R* ^2^
		*B*	*SE*	Beta			
1	(Constant)	0.0	6.6		0.0	.999	.15
	Sex, girl/boy	0.8	3.4	0.0	0.2	.807	
	Age	0.7	0.4	0.2	1.5	.142	
	Time since last needle insertion	−0.1	0.1	−0.1	−1.1	.291	
	Cortisol change^a^	0.0	0.0	0.0	−0.4	.722	
	Fear	0.3	0.1	0.4	3.4	.001	
4	(Constant)	8.7	1.9		4.6	.000	.11
	Fear	0.2	0.1	0.3	3.2	.002	

a Sample 1: directly after needle insertion. Sample 2: 30 min later.

### 3.3 Post hoc analyses of the age perspective

**Figure 2:**
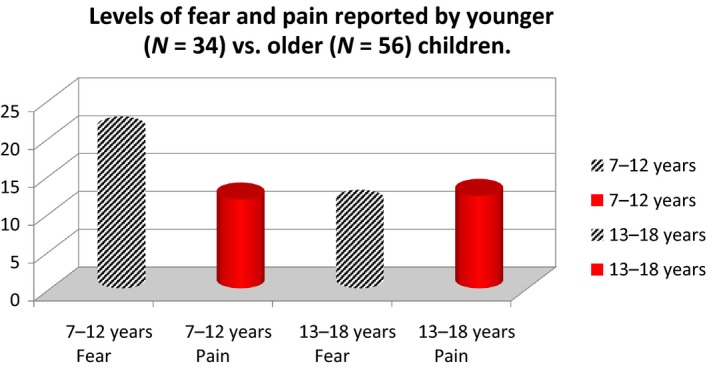
Post hoc analysis of reported fear and pain levels with regard to age

A post hoc analysis indicated that fear levels were higher than pain levels for younger children (<12 year) during the needle insertion (Table 7, Figure 2).

**Table 7:** Analyses of fear and pain levels reported with regard to age group: younger children (<12 years) and older children (≥12 years)

	VAS^a^	*N*	*SD*	Mean *SE*	*p*‐value^b^
Younger					
Fear child	20	34	25	4.232	.029
Pain child	12	34	18	3.096	
Older					
Fear child	11	56	20	2.638	.630
Pain child	12	56	14	1.920	

a Visual analogue scales 0–100 mm.

b Paired samples statistic.

In the older age group (≥12 year), the level of cortisol 30 min after the needle insertion was significantly lower than at baseline. In the younger age group, no difference was found (Table 8).

**Table 8:** Analyses of cortisol levels with regard to age group: younger children (<12 years) and older children (≥12 years)

	Mean^a^	*N*	*SD*	Mean *SE*	*p*‐value^b^
Younger					
Sample^c^ 1	131	31	115	20.683	.705
Sample 2	127	31	111	19.896	
Older					
Sample 1	214	52	140	19.452	.000
Sample 2	178	52	125	17.395	

a S‐Cortisol mmol/l.

b Paired samples statistic.

c Sample 1: directly after needle insertion – sample 2: 30 min later.

## 4 DISCUSSION

Children undergoing needle insertion with topical anaesthesia experienced fear levels as high as pain levels. Furthermore, fear and pain levels were positively associated with each other. It has previously been suggested that fear can increase the pain experience (Rhudy & Meagher, 2003) and that fear can increase during acute pain (Taddio & McMurtry, 2015). Fear was associated with children's age and pain. In the multiple regression analysis, pain and age explained 16% of the variance in fear, while fear explained 11% of the variance in pain. Therefore, the post hoc analysis was added and we found that children in the younger age group reported higher fear levels than children in the older age group; it is possible that less‐developed coping strategies and lower cognitive level contributed to this. No such difference was found for pain levels. These findings are in concordance with our earlier study of parents' proxy reports of their children's levels of fear and pain during a needle procedure (Hedén et al., 2016).

In concordance with the inverse association between age and fear, we also found a reduction of cortisol levels in the older age group (≥12 year) 30 min after needle insertion. This result may indicate a faster recovery and more developed fear‐coping strategies in older children compared with younger children. The cortisol values decreased from the first sample directly after the needle insertion to the second sample 30 min later. This suggests that the first value may not have been a true baseline value, as we had expected, but rather reflected that the pain‐related increase in cortisol may have been more rapid than anticipated. Fear of pain during the needle insertion may also have played a role with these relatively low levels of pain, thus increasing the “baseline” cortisol levels.

The major strength of this study is that children themselves are heard through self‐report of their experienced fear and pain levels. Other strengths of the study were the large study sample and the standardized model using needle insertion as a painful procedure to investigate needle fear and pain.

The fear level during needle insertion was positively correlated with the pain level. It could be asked whether it is possible for children to distinguish between the experiences of fear and pain. However, our previous studies found significant differences between children's fear and pain reports during needle procedures, which suggest that it is possible for many children to make this distinction (Hedén, Essen, Frykholm, et al., 2009; Hedén, Essen, Frykholm, et al., 2009). Nevertheless, developmental differences with age must always be taken into account. To this end and acknowledging that self‐report is generally recommended for evaluation of symptom intensity, (Twycross, Voepel‐Lewis, Vincent, Franck, & von Baeyer, 2015) we chose to include children of 7 years of age and up in this analysis.

A period effect of needle pain and fear has been suggested; (von Baeyer, Marche, Rocha, & Salmon, 2004) that is, decreasing pain and fear over time as the child gets used to the needle procedures. On the other hand, when the needle procedures are less frequent, pain and fear may increase again. In the present study, the time since last needle insertion was not associated with pain or fear, which could speak against a period effect.

## 5 LIMITATIONS

A limitation is that children with previously known and documented needs for pharmacological sedation were excluded in the randomized studies in the wider project, for ethical reasons. Thus, the results may not be representative of the children experiencing the most fear and pain.

The present study included both the placebo and the intervention group, and the results reflect fear and pain levels in connection with needle insertion in general. It could be argued that this may have introduced a bias. However, our rationale for combining the groups is that the analyses are based on the relationship between fear and pain levels, rather than the experienced intensity of fear and pain, which in our opinion is relevant for both groups. To control for this, the groups were analysed separately with regard to the relationship between fear and pain levels and we found no differences between the groups.

## 6 CONCLUSION

Fear was an important factor in children's experiences of the needle procedure. Fear levels were as high as pain levels during needle insertion under topical anaesthesia in a subcutaneously implanted port, and the levels of fear and pain were positively associated with each other.

## 7 RELEVANCE TO CLINICAL PRACTICE

Thus, when planning needle procedures in children with ongoing cancer treatment or follow‐up, it seems to be important to consider not only pain, but also fear including the phases of information, preparation and evaluating, especially in younger children. The findings may also be relevant for children with other diagnoses than cancer undergoing long‐term medical treatments including needle procedures or other painful situations.

## CONFLICT OF INTEREST

None declared.
